# MCU-i4, a mitochondrial Ca^2+^ uniporter modulator, induces breast cancer BT474 cell death by enhancing glycolysis, ATP production and reactive oxygen species (ROS) burst

**DOI:** 10.32604/or.2024.052743

**Published:** 2025-01-16

**Authors:** EDMUND CHEUNG SO, LOUIS W. C. CHOW, CHIN-MIN CHUANG, CING YU CHEN, CHENG-HSUN WU, LIAN-RU SHIAO, TING-TSZ OU, KAR-LOK WONG, YUK-MAN LEUNG, YI-PING HUANG

**Affiliations:** 1 Department of Anesthesia, An Nan Hospital, China Medical University, Tainan, 709, Taiwan; 2 Graduate Institute of Medical Sciences, Chang Jung Christian University, Tainan, 711301, Taiwan; 3 UNIMED Medical Institute, Hong Kong SAR, China; 4 Department of Emergency Medicine, China Medical University Hospital, Taichung, 404327, Taiwan; 5 Department of Cosmetic Science, Providence University, Taichung, 43301, Taiwan; 6 School of Pharmacy, China Medical University, Taichung, 404328, Taiwan; 7 Department of Anatomy, China Medical University, Taichung, 404328, Taiwan; 8 Department of Physiology, China Medical University, Taichung, 404328, Taiwan; 9 Department of Medicinal Botanicals and Healthcare, Dayeh University, Changhua, 51591, Taiwan; 10 Department of Anesthesiology, Kuang Tien General Hospital, Shalu, Taichung, 433, Taiwan

**Keywords:** BT474 cells, Breast cancer, MCU-i4, Cell death, Mitochondria Ca^2+^ uniporter (MCU)

## Abstract

**Objectives:**

Mitochondrial Ca^2+^ uniporter (MCU) provides a Ca^2+^ influx pathway from the cytosol into the mitochondrial matrix and a moderate mitochondrial Ca^2+^ rise stimulates ATP production and cell growth. MCU is highly expressed in various cancer cells including breast cancer cells, thereby increasing the capacity of mitochondrial Ca^2+^ uptake, ATP production, and cancer cell proliferation. The objective of this study was to examine MCU inhibition as an anti-cancer mechanism.

**Methods:**

The effects of MCU-i4, a newly developed MCU inhibitor, on cell viability, apoptosis, cytosolic Ca^2+^, mitochondrial Ca^2+^ and potential, glycolytic rate, generation of ATP, and reactive oxygen species, were examined in breast cancer BT474 cells.

**Results:**

MCU-i4 caused apoptotic cell death, and it decreased and increased, respectively, mitochondrial and cytosolic Ca^2+^ concentration. Inhibition of MCU by MCU-i4 revealed that cytosolic Ca^2+^ elevation resulted from endoplasmic reticulum (ER) Ca^2+^ release via inositol 1,4,5-trisphosphate receptors (IP3R) and ryanodine receptors (RYR). Unexpectedly, MCU-i4 enhanced glycolysis and ATP production; it also triggered a large production of reactive oxygen species (ROS) and mitochondrial membrane potential collapse.

**Conclusion:**

Cytotoxic mechanisms of MCU-i4 in cancer cells involved enhanced glycolysis and heightened formation of ATP and ROS. It is conventionally believed that cancer cell death could be caused by inhibition of glycolysis. Our observations suggest cancer cell death could also be induced by increased glycolytic metabolism.

## Introduction

Ca^2+^ ions are important for living cells, and their trafficking and signaling are under control through different kinds of cation channels, uniporters, and receptors [1]. Several studies have shown that regulation of Ca^2+^ signaling by modulating ion channel gating could induce cell death in cancer cells with different pathways [2–4]. A tight control of mitochondria matrix Ca^2+^ level is necessary, as a small rise could stimulate tricarboxylic acid (TCA) cycle enzymes, resulting in enhanced ATP production; on the contrary, excessive mitochondrial matrix Ca^2+^, as a result of cytosolic Ca^2+^ overload caused by deleterious agents, would result in cell death [5,6]. It has been demonstrated that constitutive endoplasmic reticulum (ER)-mitochondria Ca^2+^ transfer is necessary for normal mitochondrial functioning. For instance, Ca^2+^ released via inositol 1,4,5-trisphosphate receptors (IP3R) of ER flows into mitochondria to stimulate oxidative phosphorylation [7–9].

The molecular machinery responsible for the mitochondria to uptake Ca^2+^ from the cytosol is the mitochondrial calcium uniporter (MCU), a molecular complex residing in the inner mitochondrial membrane [10,11]. This complex comprises the MCU channel itself and accessory regulatory proteins, namely, MCU regulatory subunit β (MCUβ), essential MCU regulator (EMRE), mitochondrial Ca^2+^ uptake proteins (MICU1, 2, and 3), and mitochondrial Ca^2+^ uniporter regulator 1 (MCUR1) [12,13]. MCU is positively and negatively regulated by MCUR1 and MICU1, respectively [14].

Remarkably, MCU is highly expressed in various cancer cells including breast cancer cells, thereby increasing the capacity of mitochondrial Ca^2+^ uptake, ATP production, and cell proliferation [15]. MCU-mediated Ca^2+^ entry into the mitochondrial matrix stimulates colorectal cancer growth [16]. MCU promotes pancreatic ductal adenocarcinoma metastasis and metabolic stress resistance [17]. There is a high correlation between MCU expression and tumor growth and metastasis of triple-negative breast cancer; down-regulation of MCU attenuates tumor growth and invasiveness [18]. MCU inhibitors are therefore potential anti-cancer drugs [5]. Classical MCU inhibitors such as ruthenium red and ruthenium 360 are direct pore blockers of MCU [19,20]. A new category of MCU-inhibiting drugs was introduced recently: MCU-i4 binds to and stimulates MICU-1, and since the latter negatively regulates MCU, MCU-i4 serves as a negative modulator of MCU and inhibits Ca^2+^ uptake into the mitochondrial matrix [21]. MCU-i4 fails to inhibit mitochondrial Ca^2+^ uptake in MICU1-silenced cells or cells expressing a MICU1 mutant lacking an MCU-i4-binding site [21].

In our study, we aim to investigate the cytotoxic actions of MCU-i4 in breast cancer BT474 cells. In particular, we aim to examine how MCU-i4 modulated cytosolic Ca^2+^ homeostasis, mitochondrial functions and metabolism to cause such cytotoxicity.

## Materials and Methods

### Cell culture and materials

Fetal calf serum, Dulbecco’s modified Eagle’s medium (DMEM), and tissue culture reagents were purchased from Invitrogen Corporation (Carlsbad, CA, USA). Carbonyl cyanide-*p*-trifluoromethoxyphenylhydrazone (FCCP), 2-aminoethoxydiphenyl borate (2-APB) and cyclosporin A were from Sigma-Aldrich chemical Co. (St. Louis, MO, USA). JTV-519 and MCU-i4 were from Tocris BioScience (Bristol, UK). All other chemicals were of reagent grades and were from Sigma-Aldrich. BT474 cells were purchased from American Type Culture Collection (Manassas, VA, USA), and were cultured in RPMI-1640 medium supplemented with L-glutamine (2 mM), 10% fetal bovine serum, penicillin (100 U/mL), and streptomycin (100 μg/mL) at 37°C (98.6°F) with 5% humidified CO_2_.

### Assay of cell viability and apoptosis

BT474 cells were cultured in 96-well plates at a density of 1.5 × 10^4^ and were treated with different agents for 48 h. DMSO was added to the medium as solvent control (final concentration = 0.1%). 3-(4,5-dimethylthiazol-2-yl)-2,5-diphenltetrazolium bromide (MTT; final concentration of 0.5 mg/mL) was added to each well and further incubated for 4 h. Culture medium was discarded and DMSO (100 μL) was added to each well for another 15 min with mild shaking to dissolve precipitates. Absorbance at 595 nm was measured by an ELISA reader; absorbance was used to indicate cell viability or metabolic activities. A reduction in MTT absorbance indicates cell death, reduced cell proliferation or metabolic activities. Number of viable cells was quantified by the trypan blue exclusion method: viable cells were unstained by trypan blue and were counted with a hemocytometer. An FITC annexin V apoptosis detection kit (BioLegend, San Diego, CA, USA) and flow cytometer (BD Biosciences, San Jose, CA, USA) were used to quantify apoptosis. Caspase-9 level was measured using an ELISA kit (cat. # E-EL-H0663; Elabscience, Houston, TX, USA) following the instructions in the manufacturer’s manual.

### Microfluorimetric measurement of cytosolic Ca^2+^

Cytosol Ca^2+^ concentration was measured using fura-2 as a fluorescent probe [22]. Cells were incubated with 5 μM fura-2 AM (Invitrogen) at 37°C (98.6°F) for 1 h; cells were then washed in bath solution (mM): 140 NaCl, 2 CaCl_2_, 1 MgCl_2_, 4 KCl, 10 HEPES (pH was adjusted to 7.4 by NaOH). Intracellular Ca^2+^ release was examined in Ca^2+^-free bath solution; the latter being the same as the bath solution, except that Ca^2+^ was removed and EGTA (100 μM) was added. Cells were excited by 340 and 380 nm alternately (switching frequency = 1 Hz) by an optical filter changer (Lambda 10-2, Sutter Instruments, Novato, CA, USA). Emission was collected at 500 nm. and data were captured by a CCD camera (CoolSnap HQ2, Photometrics, Tucson, AZ, USA), which was connected to a Nikon (Tokyo, Japan) TE2000-U microscope. Data were analyzed with an MAG Biosystems Software (Sante Fe, MN, USA). Experiments were conducted at 25°C (77°F). Changes in 340/380 ratio were analyzed at a region of interest of single cells; the same experimental procedures were repeated multiple times to obtain the mean.

### Measurement of mitochondrial Ca^2+^ concentration

Microfluorimetric quantification of Ca^2+^ concentration within the mitochondrial matrix was performed using a Ca^2+^-sensitive dye Rhod-2 AM [23]. The cells were incubated with 5 μM Rhod-2 AM (Invitrogen, Carlsbad, CA, USA) at 37°C (98.6°F) for 1 h. Cells were then permeabilized and washed with a digitonin (30 μM)-containing intracellular solution, which contained (mM): 140 KCl, 8 NaCl, 1 CaCl_2_, 1 MgCl_2_, 1.85 EGTA, 10 HEPES, and 8 MgATP (KOH was used to adjust pH to 7.25). Free [Ca^2+^] in this intracellular solution was calculated to be 114 nM. Excitation wavelength was 540 nm and emission wavelength was 605 nm. Images were captured by a CCD camera (CoolSnap HQ2, Photometrics, Tucson, AZ, USA) connected to an inverted microscope (Nikon TE 2000-U). An MAG Biosystems Software (Sante Fe, MN) was used for analysis. All experiments were performed at room temperature (25°C) (77°F).

### Measurement of mitochondrial membrane potential

Mitochondrial membrane potential was measured by a mitochondrial membrane potential assay kit (#12664; Cell Signaling, Danvers, MA, USA) as described in a previous report [24]. The cells were plated at a density of 5 × 10^4^ cells per well to settle overnight. The cells were treated with DMSO or other agents for 24 h. JC-1 (2 μM) was added to each well for 30 min. Fluorescent emission was measured by a Varioskan LUX multimode microplate reader (Thermo Fisher Scientific, Waltham, MA, USA). Excitation wavelength was at 485 nm and dual emission wavelengths were at 520 and 590 nm. Mitochondrial membrane potential was calculated as the ratio RFU of red emission (590 nm)/RFU of green emission (520 nm).

### Reactive oxygen species (ROS) assay

ROS was measured as described in a previous report [25]. The cells were treated with various agents for 4 h, and were then incubated in serum-free DMEM supplemented with 2,7-dichlorodihydrofluorescein diacetate (DCFH2-DA, 20 μM, Sigma, St. Louis, MO, USA) at 37°C (98.6°F) for 30 min in darkness. Cells were then washed, trypsinized at 37°C for 3 min, and washed again three more times in phosphate-buffered saline using centrifugation. The cells were then dispersed in phosphate-buffered saline, put in polystyrene tubes for FACS (fluorescence-activated cell sorting). The samples (1 × 10^5^ cells/sample) were analyzed by an FACS Canto flow cytometer system (BD Biosciences, San Jose, CA, USA). Data analysis was performed with the aid of a BD FACSDIVA™ software (BD Biosciences).

### Assay of ATP content

Cellular ATP content was quantified as described in a previous report [26]. The cells were seeded in 10-cm dishes at a density of 6 × 10^6^ cells per dish and treated with DMSO or 30 μM MCU-i4 for 24 h. The cells were subsequently trypsinized and cell viability was quantified by the trypan blue exclusion method. The cells were sonicated (33 Hz, 90 s) in ice bath, centrifuged (10,000 g, 10 min) and 30 μL supernatant was taken for ATP quantification using an ATP assay kit (catalogue # E-BC-K57-M; Elabscience, Houston, TX, USA). Samples were analyzed by a BioTek Epoch 2 microplate spectrophotometer (Winooski, VT, USA).

### Assay of glycolytic activity

Quantification of lactate was used as an indicator of glycolysis [27]. The cells were seeded in 96-well plates at a density of 1 × 10^4^ cells/per well overnight. The cells were then treated with DMSO or 30 μM MCU-i4 for 3 h, and 5 μL supernatant was taken for lactate quantification using a glycolysis assay kit (catalogue # ECGL-100; BioAssay Systems, Hayward, CA, USA), and samples were analyzed by a BioTek Epoch 2 microplate spectrophotometer (Winooski, VT, USA).

### Statistical analysis

Statistical analysis and graphing were performed using Origin8.5 (OriginLab, MA, USA). Results are presented as means ± standard error of mean (S.E.M.). Paired or unpaired Student *t*-test was employed where appropriate to compare two groups. When multiple groups were analyzed, ANOVA and the Tukey’s HSD *post-hoc* test were used. Statistical significance was considered to be reached if the *p*-value is less than 0.05.

## Results

### MCU-i4 induced apoptotic cell death

**Figure 1:**
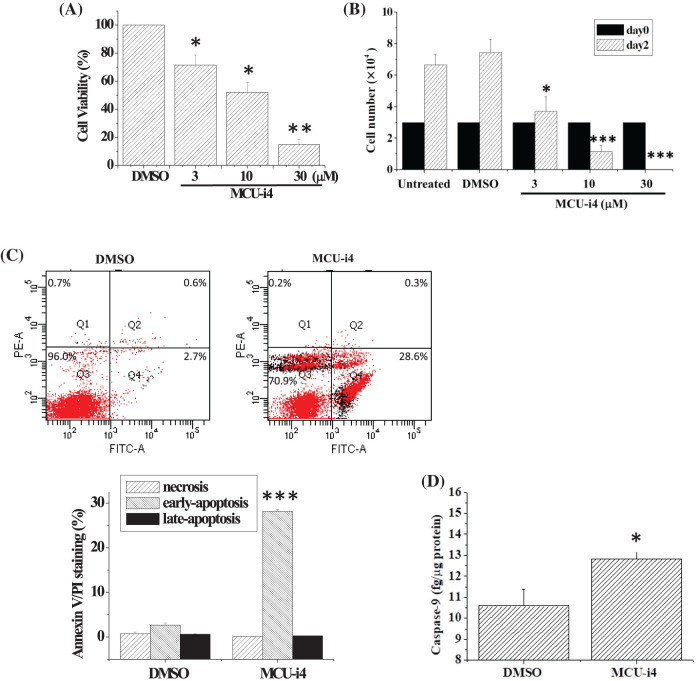
MCU-4 decreased cell viability and caused apoptosis. (A) Cells were treated with different concentrations of MCU-i4 for 2 days before the MTT assay was used to determine cell viability. (B) Cells, initially seeded on 24-well plates at a density of 3 × 10^4^/well, received no vehicle or drug (untreated) or were treated with DMSO (vehicle) or different concentrations of MCU-i4 for 2 days, and viable cells were counted by the trypan blue exclusion method. (C) Cells were treated with DMSO or 30 μM MCU-i4 for 1 day and examined for apoptosis. (D) Cells were cultured on 6-cm wells at a density of 5 × 10^5^/well (in order to have sufficient protein harvest for caspase-9 determination), treated with DMSO or 30 μM MCU-i4 for 2 days, and examined for the level of caspase-9. Results are mean ± SEM from 3–4 independent experiments. *p < 0.05, **p < 0.01, ***p < 0.001 different from the DMSO control.

Treatment of BT474 cells with MCU-i4 (3–30 μM) for 2 days resulted in a concentration-dependent decrease in cell viability (Fig. 1A). Since the MTT assay shown in Fig. 1A could not discriminate between cell death and reduced cell proliferation, we used the trypan blue exclusion method to quantitate the number of viable cells (Fig. 1B). Cell proliferation was suppressed by 3 μM MCU-i4, while higher concentrations (10–30 μM) concentration-dependently caused cell death. We investigated whether cell death was necrotic or apoptotic. There was a 10-fold increase in annexin-positive/propidium iodide-negative cells (Q4), suggesting early apoptosis had taken place (Fig. 1C). Caspase-9 has been known as a marker of apoptosis [28]. Consistently, MCU-i4 treatment also resulted in a moderate increase in the level of caspase-9 (Fig. 1D).

### Effects of MCU-i4 on cytosolic Ca^2+^ fluxes

**Figure 2:**
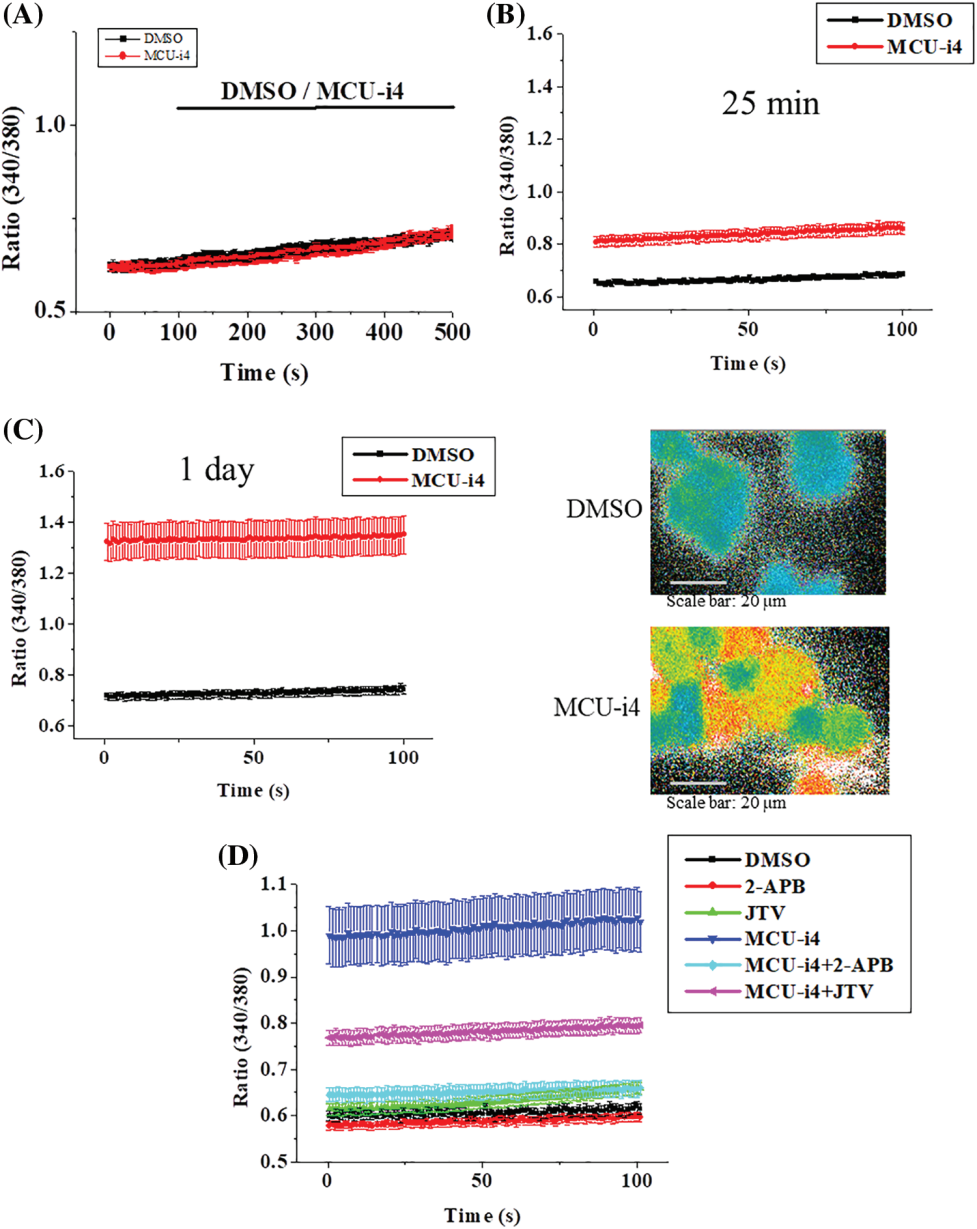
Effects of MCU-i4 (30 μM) on Ca^2+^ fluxes. (A) Cells in Ca^2+^-containing solution were exposed to DMSO (vehicle control) or MCU-i4. (B) Cells were pretreated with DMSO or MCU-i4 for 25 min in a Ca^2+^-containing solution and then subject to microfluorimetric measurement. (C) Cells were cultured in the presence of DMSO or MCU-i4 for 1 day, loaded with fura-2, bathed in Ca^2+^-containing solution, and then subject to microfluorimetric measurement. The right panels show pseudocolor images (low ratio being blue to high ratio being red) of fluorescent ratio analysis of DMSO-and MCU-i4-treated cells. (D) Cells were pretreated with DMSO or MCU-i4 (in the absence or presence of 30 μM JTV-159 or 30 μM 2-APB) for 25 min in Ca^2+^-free solution and then subject to microfluorimetric measurement. For (B)–(D), there are significant (*p* < 0.001) differences between the DMSO and MCU-i4 groups at all time points. For (D), there are significant (*p* < 0.001) differences between the MCU-i4 group and MCU-i4 plus JTV-159 group or MCU-i4 plus 2-APB group at all time points. Results are mean ± S.E.M.; each group had 14–57 cells from 3 independent experiments.

We investigated whether MCU-i4 affected cytosolic Ca^2+^ levels. MCU-i4 did not cause an immediate elevation in cytosolic Ca^2+^ concentration [Ca^2+^]_i_ (Fig. 2A). We then investigated whether a prolonged MCU-i4 pre-treatment (25 min) would affect the Ca^2+^ level. The cells in Ca^2+^-containing bath solution were treated with DMSO or MCU-i4 for 25 min (kept in the dark to avoid photobleaching) prior to microfluorimetric measurement. An elevated Ca^2+^ baseline was observed in the MCU-i4-treated cells (Fig. 2B). Treatment with MCU-i4 for 24 h (followed by fura 2 loading and microfluorimetric measurements) also resulted in an elevated Ca^2+^ baseline, suggesting prolonged MCU-i4 treatment raised Ca^2+^ concentration in the cytosol (Fig. 2C). We repeated the above protocol (25 min) in Ca^2+^-free bath solution (Fig. 2D). An elevated Ca^2+^ baseline was again observed in the MCU-i4-treated cells, suggesting the raised Ca^2+^ concentration in the cytosol was in part due to Ca^2+^ release. Inhibition of inositol 1,4,5-trisphosphate receptors (IP3R) by 2-APB strongly suppressed the elevation of Ca^2+^ baseline, while inhibition of ryanodine receptors (RYR) by JTV-519 only mildly alleviated it, suggesting the Ca^2+^ leak was mainly via IP3R and in part via RYR.

### MCU-i4 reduced mitochondrial matrix Ca^2+^ level

**Figure 3:**
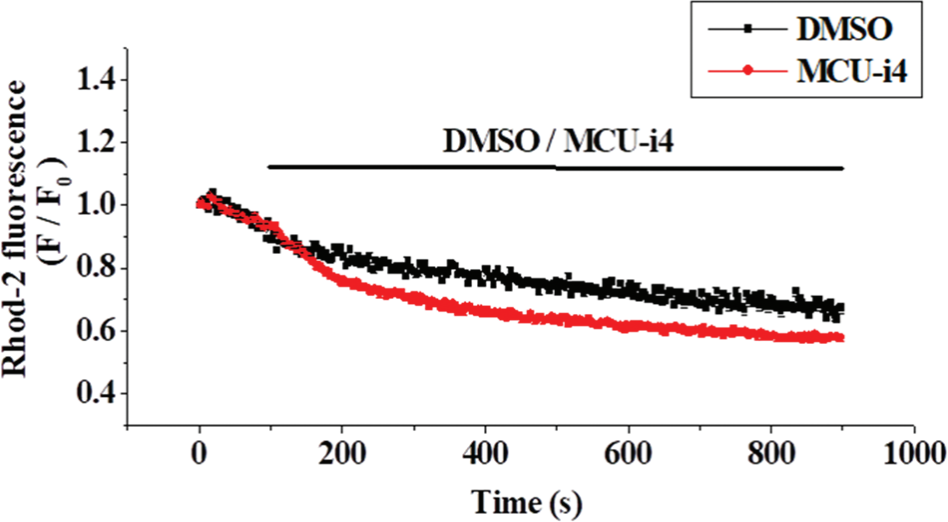
MCU-i4 (30 μM) caused a decrease in mitochondrial Ca^2+^ level. Cells were loaded with Rhod-2 and permeabilized as described in the methods section. Cells were then treated with DMSO or MCU-i4. Changes in mitochondrial Ca^2+^ level are quantified as fluorescence/fluorescence at time zero (F/F_0_). There are significant differences between the DMSO control and the MCU-i4 group after 171 s (*p* < 0.05). Results are mean ± SEM; each group had 26–31 cells from 3 independent experiments.

Ca^2+^ release from intracellular Ca^2+^ store caused elevation in cytosolic Ca^2+^ level under inhibition of mitochondrial Ca^2+^ uptake by MCU-i4 (Fig. 2). To show that inhibition of mitochondrial Ca^2+^ uptake by MCU-i4 led to a decrease in mitochondrial Ca^2+^ concentration, we used Rhod-2, a fluorescent probe for Ca^2+^ concentration in the mitochondrial matrix (Fig. 3). In the control where DMSO was added, there was a slow decrease in fluorescence which was due to inevitable photobleaching of the fluorescent dye; addition of MCU-i4 caused an immediate and persistent decrease in fluorescence when compared to the control, indicating a decrease in mitochondrial matrix Ca^2+^ concentration. This result, together with the data in Fig. 2, suggest that upon MCU-i4 inhibition of mitochondrial Ca^2+^ uptake, Ca^2+^ released from Ca^2+^ store failed to enter mitochondria and thus “spilled over” in the cytosol.

### MCU-i4 enhanced glycolysis and production of ATP and ROS

**Figure 4:**
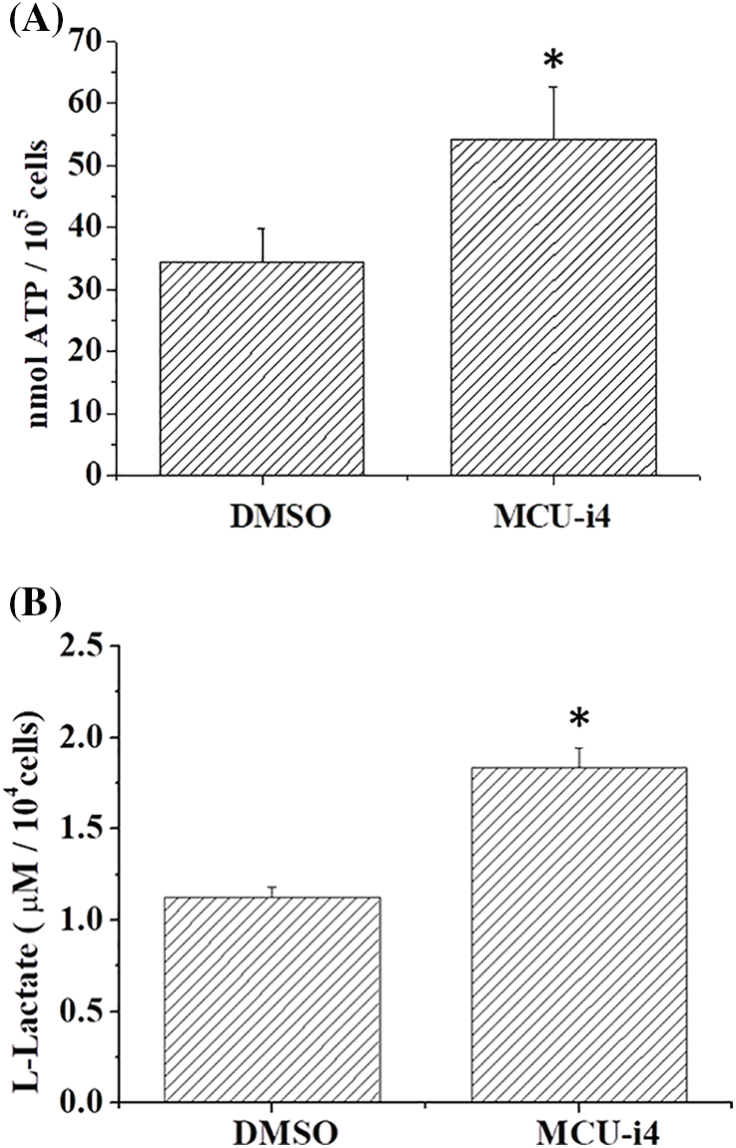
MCU-i4 treatment increased ATP and lactate production. (A) Cells were treated with DMSO or 30 μM MCU-i4 for one day and then subject to ATP quantification. (B) Cells were treated with DMSO or 30 μM MCU-i4 for 3 h and then subject to lactate quantification. Results are mean ± SEM from 4 independent experiments. Significantly different from the DMSO control *p < 0.05.

**Figure 5:**
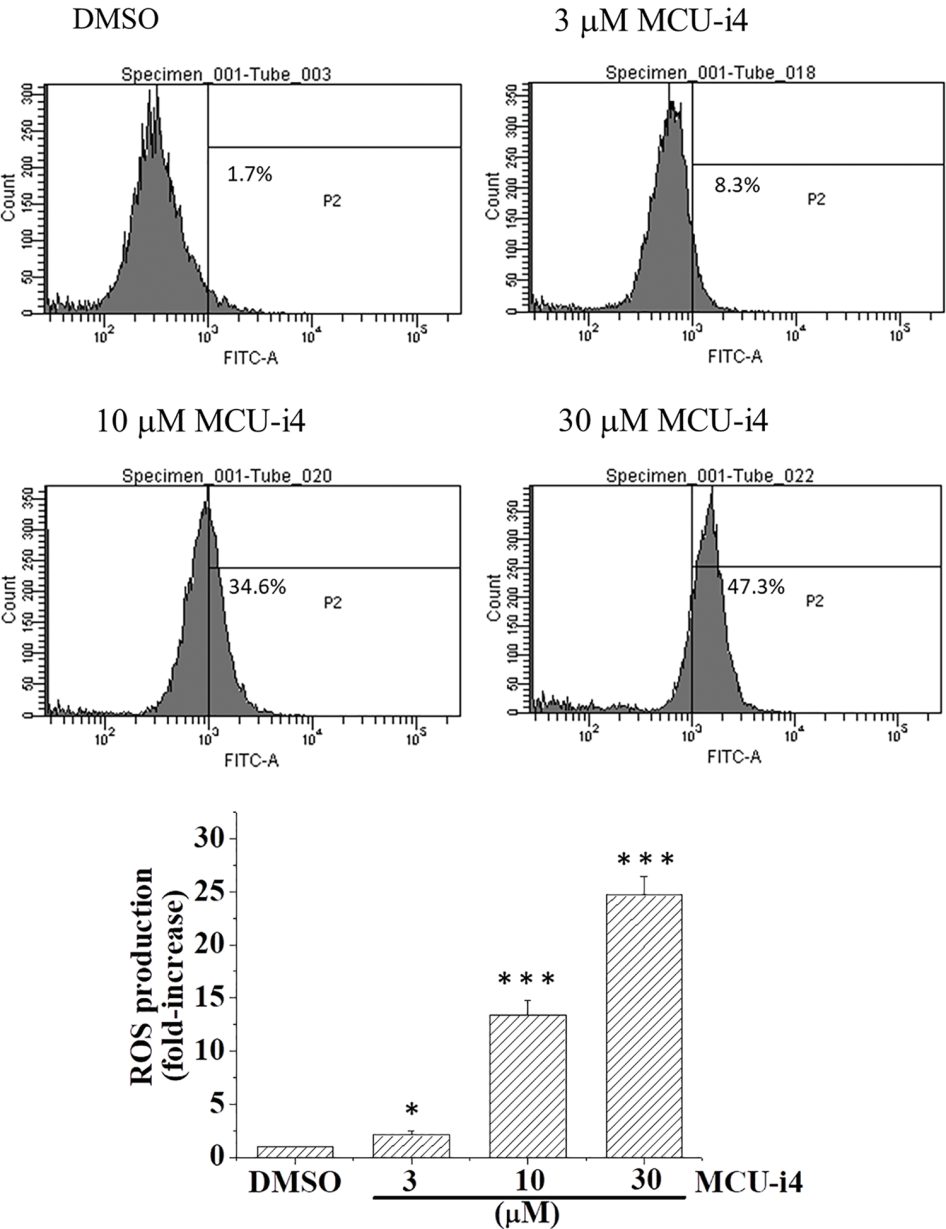
MCU-i4 triggered ROS formation. Cells were treated with DMSO or different concentrations of MCU-i4 for 4 h and then subject to ROS measurement using flow cytometry. Results represent mean ± SEM from 6 independent experiments. *p < 0.05, ***p < 0.001 significantly different from the DMSO control.

We next examined whether MCU-i4-induced lowered mitochondrial matrix Ca^2+^ level would affect ATP production. MCU-i4 treatment for 24 h resulted in a 51.9 ± 5.8% decrease in viable cell count (trypan blue exclusion test) but only moderately reduced ATP production by 23.9 ± 9.8%. When ATP production was normalized by the number of viable cells, MCU-i4 treatment significantly enhanced ATP production (Fig. 4A). Since lowered mitochondrial matrix Ca^2+^ level was not compatible with increased mitochondrial ATP production, we examined whether increased ATP production resulted from increased glycolytic activities. As shown in Fig. 4B, MCU-i4 caused a 1.6-fold elevation in secreted lactate concentration, which indicated an increase in glycolysis. Whether MCU-i4 elicited ROS formation was next examined. As shown in Fig. 5, treatment of cells with MCU-i4 for 4 h resulted in large production of ROS.

### Effects of MCU-i4 on mitochondrial membrane potential

**Figure 6:**
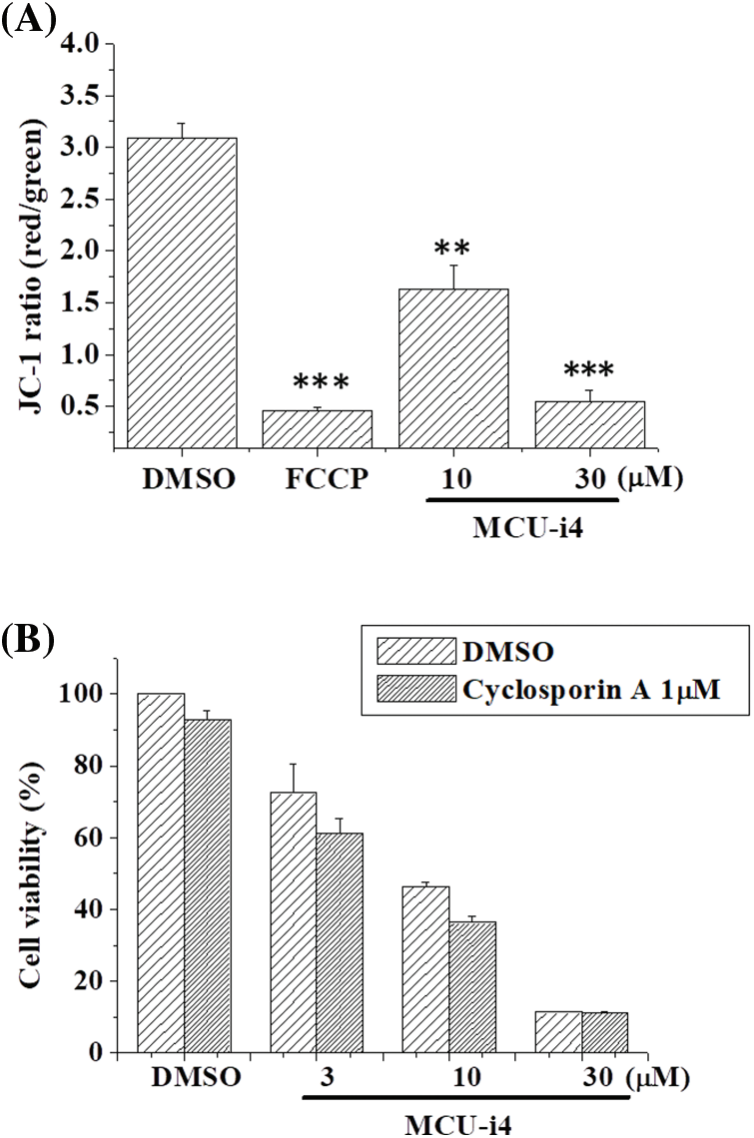
MCU-i4 caused mitochondrial membrane depolarization but decreased viability was not rescued by cyclosporin A. (A) Cells were treated with 10–30 μM MCU-i4 for one day and then subject to mitochondrial membrane potential measurement. FCCP (4-h treatment) was used as a positive control for membrane potential collapse. (B) Cells were treated with different concentrations of MCU-i4 in the absence or presence of 1 μM cyclosporin A for 2 days before MTT assay was employed to quantify cell viability. Results are mean ± SEM from 3 independent experiments. **p < 0.01, ***p < 0.001 significantly different from the DMSO control.

Using JC-1 as a fluorescent probe of mitochondrial membrane potential, we examined if MCU-i4 would depolarize mitochondrial potential. MCU-i4 at 10 μM caused marked depolarization whilst at 30 μM it caused collapse of mitochondrial membrane potential to an extent comparable to that caused by FCCP (Fig. 6A). Since mitochondrial membrane potential collapse could be a result of mitochondria permeability transition pore (MPTP) opening and that cyclophilin D is an integral part of the MPTP, we examined if cyclosporin A (an MPTP inhibitor by interacting with cyclophilin D) could reduce MCU-i4 cytotoxicity. However, MCU-i4-inflicted cell death was not prevented by cyclosporin A (Fig. 6B).

## Discussion

Classical MCU inhibitors (direct pore blockers) such as ruthenium complexes (ruthenium red and Ru360) have been shown to induce apoptosis in kidney tubular cyst cells and colon carcinoma HCT-116 cells [29,30]. By contrast, they inhibit apoptosis in colonocytes, mammary gland adenocarcinoma cells, neuroblastoma cells, and podocytes, by preventing mitochondrial Ca^2+^ overload [31–34]. The factors governing whether these ruthenium-related compounds prevent or induce apoptosis are unclear. The antibiotics minocycline and doxycycline also cause MCU inhibition, which may lead to their anti-cancer activities [5,35–37]. MCU-i4 differs from the classical MCU pore blockers: it binds to MICU1 and is therefore a negative modulator of MCU [21]. We here provided data to show how MCU-i4 caused apoptotic death in cancer cells.

In our report, we showed MCU-i4 decreased mitochondrial matrix Ca^2+^ level. This, however, did not lead to the expected reduction of ATP production but instead caused a moderate increase in ATP production. One explanation is that the reduction in mitochondrial matrix Ca^2+^ level was too mild to cause a significant drop in ATP production, whilst persistent cytosolic Ca^2+^ rise (Fig. 2) enhanced glycolytic production of ATP. This is supported by our findings that MCU-i4 caused increases in glycolytic activity and ATP production (Fig. 4). Our data are therefore in concordance with previous observations showing that Ca^2+^ stimulates glycolysis [38,39]. Our study showing that MCU-i4 caused increased ATP production and apoptotic (instead of necrotic) cell death (Fig. 1) is reminiscent of previous reports demonstrating that cells undergoing apoptosis had raised levels of ATP. For instance, cerebellar granule cells undergoing apoptosis had enhanced ATP production derived from both oxidative phosphorylation and glycolysis [40]. HeLa, PC12 and U937 cells had increased cytosolic ATP levels when they underwent apoptosis; inhibition of glycolysis abolished apoptosis [41]. In isolated hypoxic rat cardiac myocytes, cell death shifted from necrosis to apoptosis when cellular ATP level was raised by increasing glucose concentration in the medium [42]. Therefore, lack and abundance of ATP favor, respectively, necrosis and apoptosis [43].

In heart failure, disturbed Ca^2+^ handling reduces mitochondrial Ca^2+^ uptake and results in oxidative stress in cardiomyocytes [44]. How ROS formation was raised in MCU-i4-treated BT474 cells was uncertain. It might be partly due to heightened metabolism: increased cytosolic Ca^2+^ level enhanced glycolysis (see above; Figs. 2 and 4), with pyruvate increasingly fueling the Kreb’s cycle and oxidative phosphorylation. ROS may activate hypoxia-inducible factor 1-α, which further promotes glycolysis [45]. ROS can elicit further ROS formation from proximal mitochondria, a term coined ROS-induced ROS release [46,47]. This may account for the substantial amount of ROS formation in MCU-i4-treated BT474 cells. Thus, ROS formation, together with persistent cytosolic Ca^2+^ overload (see below), may eventually lead to apoptotic cell death (Fig. 1C). The latter was also evidenced by the increase in caspase-9 level, an indicator of mitochondria-dependent apoptosis (Fig. 1D). Reduced mitochondrial Ca^2+^ level may sensitize cytotoxicity by apoptotic signals. Remarkably, a reduction in mitochondrial Ca^2+^ level by chelation or Ru360 potentiates cytotoxicity induced by tumor necrosis factor (TNF)-related apoptosis-inducing ligand (TRAIL) in apoptosis-resistant tumor cells [48].

One of the lethal causes of MCU-i4 is likely cytosolic Ca^2+^ overload, as excessive and persistent Ca^2+^ elevation activates multiple phospholipases, proteases, and caspases [49]. As constitutive ER-mitochondria Ca^2+^ transfer is necessary for normal mitochondrial functioning such as oxidative phosphorylation [7–9], blockade of Ca^2+^ influx into the mitochondrial matrix by MCU-i4 resulted in sustained cytosolic Ca^2+^ elevation. Our data suggest that Ca^2+^ released from ER was mainly via IP3R and in part RYR. Although MICU1 is the known target of MCU-i4, the possibility that MCU-i4 targets on other protein molecules involved in cell metabolism, and hence inflicts cell death, could not be ruled out. Future work to identify possible off-targets is warranted.

Raised mitochondrial Ca^2+^ levels and ROS are strongly implicated in mitochondrial permeability transition pore (MPTP) opening and consequent mitochondrial membrane potential collapse [50]. Here we show that although MCU-i4 caused a mild reduction in mitochondrial Ca^2+^, the drastic ROS production it elicited might have overridden and sufficed to cause mitochondrial membrane potential collapse (which was to the same extent as elicited by FCCP). Ruthenium-related compounds reportedly do not alter mitochondrial membrane potential and thus serve as more selective probes for the MCU [51]. The ability of MCU-i4 to decrease mitochondrial membrane potential limits its selectivity but endows it with anti-cancer activities. Given that cyclophilin D is an integral part of the MPTP, the observation that MCU-i4 caused mitochondrial membrane potential collapse but its cytotoxicity was not alleviated by cyclosporin A (which binds to cyclophilin D and thus inhibits MPTP), appears intriguing. A possible explanation for this is that the MPTP opening caused by MCU-i4 was partly independent of cyclophilin D. To support this notion, evidence comes from observations that cardiomyocytes and embryonic fibroblasts from cyclophilin D knock-out mice still exhibited MPTP opening, albeit to a lesser extent than that of the wild-type counterparts [52,53].

We have presented evidence that MCU-i4 enhanced glycolysis and ATP production (Fig. 4). MCU-i4 also caused mitochondrial dysfunction (Fig. 6A), which might lead to decreased oxygen consumption. Therefore, it is likely that MCU-i4-treated BT474 cells consumed less oxygen when compared to untreated cells. A limitation of our study was the lack of data on oxygen consumption rate, which would warrant further investigation.

## Conclusion

MCU-i4 inhibited mitochondrial Ca^2+^ uptake and thus caused cytosolic Ca^2+^ overload due to continuous ER Ca^2+^ release. Increased glycolysis, ATP production, and ROS burst were followed by mitochondrial membrane potential collapse and eventually apoptotic death of BT474 cells. It is conventionally believed that cancer cell death could be caused by inhibition of glycolysis [54]. Our observations suggest cancer cell death could also be induced by increased glycolytic metabolism. The cytotoxic mechanisms of MCU-i4 may shed light on future investigations into the use of anti-cancer drugs that target the MCU.

## Data Availability

Data that support findings of this study are available from the corresponding author upon reasonable request.

## Abbreviations

MCU: Mitochondrial Ca^2+^ uniporter
ER: Endoplasmic reticulum
IP3R: Inositol 1,4,5-trisphosphate receptors
RYR: Ryanodine receptors
ROS: Reactive oxygen species
